# Mechanical behaviour of fluid-lubricated faults

**DOI:** 10.1038/s41467-019-09293-9

**Published:** 2019-03-20

**Authors:** C. Cornelio, E. Spagnuolo, G. Di Toro, S. Nielsen, M. Violay

**Affiliations:** 1 Laboratory of Experimental Rock Mechanics (LEMR), École Polytechnique Fédérale de Lausanne (EPFL), Station 18, Lausanne, CH-1015 Switzerland; 2 Istituto Nazionale di Geofisica e Vuolcanologia (INGV), Via di Vigna Murata, 605, Rome, 00143 Italy; 3 Dipartimento di Geoscienze, Università degli Studi di Padova, via G. Gradenigo, 6, Padova, 35131 Italy; 4 Department of Earth Sciences, University of Durham, Stockton Road, Durham, DH1 3LE UK

## Abstract

Fluids are pervasive in fault zones cutting the Earth's crust; however, the effect of fluid viscosity on fault mechanics is mainly conjectured by theoretical models. We present friction experiments performed on both dry and fluid-permeated silicate and carbonate bearing-rocks, at normal effective stresses up to 20 MPa, with a slip-rate ranging between 10 μm/s and 1 m/s. Four different fluid viscosities were tested. We show that both static and dynamic friction coefficients decrease with viscosity and that dynamic friction depends on the dimensionless Sommerfeld number (*S*) as predicted by the elastohydrodynamic-lubrication theory (EHD).Under favourable conditions (depending on the fluid viscosity (*η*), co-seismic slip-rate (*V*), fault geometry (*L*/*H*_*0*_^2^) and earthquake nucleation depth (∝*σ*_eff_)), EHD might be an effective weakening mechanism during natural and induced earthquakes. However, at seismic slip-rate, the slip weakening distance (*D*_c_) increases markedly for a range of fluid viscosities expected in the Earth, potentially favouring slow-slip rather than rupture propagation for small to moderate earthquakes.

## Abstract (web summary)

The effect of fluid viscosity on fault mechanics is mainly conjectured by theoretical models. Here, the authors present experimental data from rock friction experiments, showing both static and dynamic friction coefficients to decrease with viscosity and dynamic friction to depend on the Sommerfeld number.

## Introduction

Fluids with variable composition (gas, water, brine, hydrocarbon seepage, wet gouge and frictional melt), rheology and physical state are pervasive within active tectonic faults. The viscosity of such fluids may vary over seven orders of magnitude, from 10^−4^ Pa s for liquid water to 10^3^ Pa s for silicic melts at high temperature^1–3^. Elastohydrodynamic lubrication (EHD), or the weakening induced by overpressure generated by the shearing of a thin viscous fluid between two subparallel and rough surfaces^4^, has been recognised for a long time in industrial processes^5,6^. EHD has also been invoked to explain the dramatic reduction of friction during earthquake slip in the presence of fluids^7,8^. However, up to date, the possible triggering of EHD in natural faults relies on theoretical models only, and was not tested at deformation conditions typical of seismic faulting. Furthermore, the experimental studies investigating fluid-rock interaction almost exclusively considered water^9–11^ as the fluid at both subseismic and seismic slip-rates^12^.

For sake of simplicity, tectonic faults can be described at seismogenic depths as rough surfaces separating two solids, which are in contact at a number of asperities^13^, which represent only a fraction of the total fault area, and can be filled by fluids and gouges. During fault sliding and depending on how the normal stress is partitioned between the asperities and the fluid, three different regimes can been distinguished^4,14^: the boundary lubrication regime (BL), where the normal stress is supported by solid–solid contacts; the fully lubricated regime (EHD), where the normal stress is supported by interstitial fluid; and the mixed lubrication regime (ML), where the normal stress is supported both by the solid–solid contacts and the fluid. The transition between these three regimes is controlled by the Sommerfeld number^4^ $S = \frac{{6\eta VL}}{{\left( {H_0} \right)^2\sigma _{{\mathrm{eff}}}}}$, where *V* is the slip-rate, *η* is the lubricant dynamic viscosity at the estimated mean temperature^14^ of the slipping zone (defined as the zone where deformation is highly localised), *L* is the characteristic length over which the fluid pressure changes (related to the wavelength of the asperities), *H*_0_ is the initial average gap between the asperities (related to the height of the asperities) and *σ*_eff_ is the effective normal stress (*σ*_eff_ = *σ*_*n*_ − *P*_*f*_, where *σ*_*n*_ is the normal stress and *P*_*f*_ is the fluid pressure).

Recently, Bayart et al.^15,16^ provided evidence of the influence of fluid viscosity by performing laboratory stick-slip experiments as an analogue of seismic events^17^. The setup consisted of poly-methylmethacrylate (PMMA) slabs lubricated by a film of viscous fluid (silicone and hydrocarbon oils). The presence of the fluid resulted in a smaller static friction with respect to that under room-humidity conditions. However, the fracture energy (i.e., the energy dissipated by crack propagation) increased in the presence of the fluid and was independent of the lubricant viscosity but dependent of the lubricant composition.

Here, based on experimental and geological evidence, we discuss the effect of the viscosity of a fluid sandwiched between two rock slip surfaces at slip-rates characteristic of either earthquake nucleation (slip-rate from μm/s to mm/s)^18^ and propagation (mm/s to m/s)^19,20^ and, in general, at slip-rates at which the ML and the EHD regimes might be activated.

## Results

### Experimental protocol

We performed 36 experiments with the rotary machine SHIVA^21^ (INGV, Rome) on full cylinders (diameter *D* = 50 mm) of Westerly Granite and Carrara Marble either in the presence of a liquid lubricant or under room-humidity conditions. Pore fluid experiments were performed under drained conditions (a membrane pump maintained a constant fluid pressure of *P*_*f*_ = 3 MPa), preventing macroscopic fluid pressurisation inside the vessel^9^. The experiments were performed by imposing a slip acceleration of 6.5 m s^−2^ to the samples up to a target slip-rate (*V*), followed by deceleration to *V* = 0 m/s. The target *V* ranged from 0.01 to 1000 mm/s, slip distance (*U*) from 0.1 to 4 m and imposed (*σ*_*n*_) were up to 23 MPa (Supplementary Table 1). Westerly Granite and Carrara Marble were selected because (1) of their low porosity (<2% measured using the triple-weighing method^22^), which limited fluid diffusion through the rock matrix out of the sliding surface during the experiments, (2) of their very small grain size and homogeneity and (3) because typical rocks of the seismogenic continental crust^23^.

The sliding surfaces of all the samples were roughened by using 120 SiC abrasive paper (*H*_0_ ~ RMS = 7–13 μm, for Carrara Marble and Westerly Granite, respectively, measured with optical 3D profilometer, Contour GT-I 3D-Optical Microscope, Bruker). We consider the perimeter of our sample (0.157 m) as the maximum characteristic wavelength (i.e., the largest possible wavelength), due to the periodicity of rotation of the rock cylinders.

### Fluids viscosity

Various viscosities of the lubricant were obtained by mixing different volume proportions of distilled water to 99.9% glycerol. Glycerol (1, 2, 3-propanetriol) is a Newtonian, water-soluble, colourless fluid, which is stable under most conditions due to its high flash and fire points (177 and 207 °C, respectively), which prevents phase changes and fire hazards when exposed to high temperatures due to frictional heating. The viscosities at room temperature were 1.002 mPa s (pure distilled water), 10.8 mPa s (60 wt% glycerol), 109.2 mPa s (85 wt% glycerol), and 1226.6 mPa s (99.9% pure glycerol). The temperature increase with increasing slip due to frictional heating during the experiments is expected to lower the lubricant viscosity. The increase in the bulk temperature on the shearing surfaces was estimated by using a 2D time-dependent model for heat diffusion. We assumed that all the mechanical energy was converted into heat (because wear products were almost negligible in the fluid lubricated regime) and we neglected heat losses by radiation and fluid convection (the liquid was inside a vessel). As a consequence, half the instantaneous heat flow-rate *Q*(*r*,*t*) = 0.5 × *τ*(*t*) × *V*(*r*, *t*), where *r* is the radial distance from the centre of the sample, *τ* is the shear stress, was applied as a Neumann boundary condition^24^ to the edge of the model, which simulates the slip surface. Then, the estimated temperature at *r* = 2/3 *R* where *R* = 25 mm is the external radius of the sample, was used to correct the fluid viscosity following the empirical formula proposed by Cheng^25^ and to compute *S* (Supplementary Figure 8 and Supplementary Table 5 for detail on the thermal model).

### Mechanical results

**Fig. 1:**
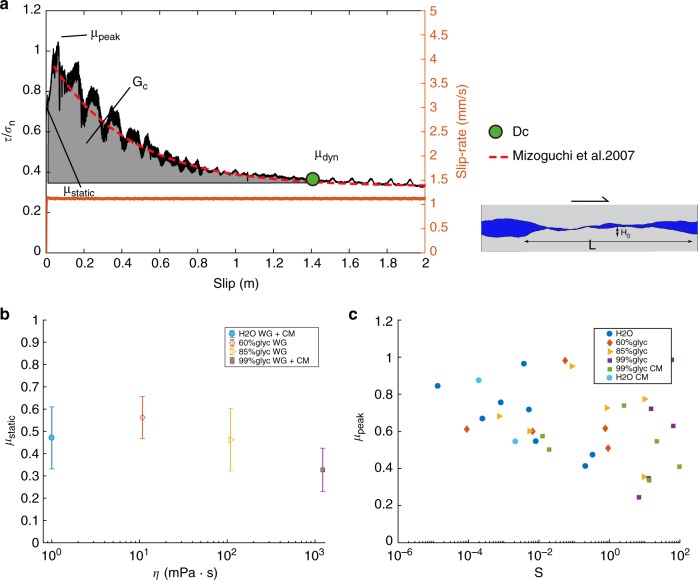
Apparent friction coefficient vs. slip, friction coefficients vs. viscosity, and Sommerfeld number. Experiments were performed at an acceleration of 6.5 m s^−2^ and effective normal stress *σ*_eff_ up to 20 MPa, under the following environmental and hydraulic conditions: 100% water (H_2_O, blue in colour dots), 60% glycerol/40% water (orange diamonds), 85% glycerol/15% water (yellow triangles), and pure glycerol (99% glycerol, purple squares). **a** Apparent friction coefficient vs. slip for experiment S1315 performed at *σ*_eff_ = 10 MPa in the presence of mixture 60% glycerol/40% water. *μ*_static_, *μ*_peak_, *μ*_dyn_, *G*_c_, and *D*_c_ are represented. The apparent friction coefficient is fitted following the exponential decay function proposed by Mizoguchi et al.^26^: $\mu = \mu _{\mathrm{dyn}} + \left( {\mu _{\mathrm{peak}} - \mu _{\mathrm{dyn}}} \right)e^{\ln \left( {0.05} \right)\,U/D_{\mathrm{c}}}$ (red line). The *G*_c_ is define as $G_{\mathrm{c}} = \mathop {\smallint }\limits_0^{D_{\mathrm{c}}} \tau \,du$ where the weakening distance *D*_c_ is the displacement over which *μ*_dyn_ is 95% of (*μ*_peak_ − *μ*_dyn_) (Mizoguchi et al.^26^). **b** Static friction coefficient vs. viscosity *η*. The error bar indicate the standard deviation from the reported average values. In the semi-logarithmic diagram, the static friction coefficient slightly decreases linearly with increasing *η* (all values are reported in Supplementary Table 1). **c** Peak friction coefficients vs. Sommerfeld number

The apparent friction coefficient (*μ*) is the ratio of the shear stress to the effective normal stress. For both rock types, at the beginning of the experiments, the shear stress acting on the fault increased until the static friction coefficient *μ*_static_ was overcome and slip initiated (Fig. 1). Immediately after slip initiation, a further increase in *μ* was observed (indicating slip-strengthening behaviour) until a peak friction *μ*_peak_ was achieved at *V* ~ 1 mm/s when the target *V* was higher than 1 mm/s. Subsequently, *μ* decreased with slip to a minimum and approximately steady-state dynamic friction coefficient (*μ*_dyn_) during a transient slip weakening phase over a slip distance (*D*_c_)^26^ (Fig. 1a). In our experiments, the achievement of this so called steady-state dynamic friction coefficient condition requires slips of several millimetres to tens of centimetres depending on the applied effective normal stress^2^, though steady-state conditions might not ever be achieved in nature^27^. Under the same acceleration conditions, the magnitude of the friction drop (*μ*_peak_ − *μ*_dyn_) increased with *V*. We studied the dependency of *μ*_static_ and *μ*_peak_ with *η* and S, respectively, for all the experiments (Fig. 1). Independently of the rock type, *μ*_static_ slightly decreased from ~0.55 at *η*_0_ = 10.8 mPa s to ~0.33 at *η*_0_ = 1226 mPa s (Fig. 1b). Instead, the *μ*_peak_ was 0.651 ± 0.222 (highly scattered) over the entire range of *S* (Fig. 1c), had no significant correlation with either *V* (Supplementary Figure 5) or *η*, and its average value was similar to the *μ*_peak_ obtained under room-humidity conditions^28^.

**Fig. 2:**
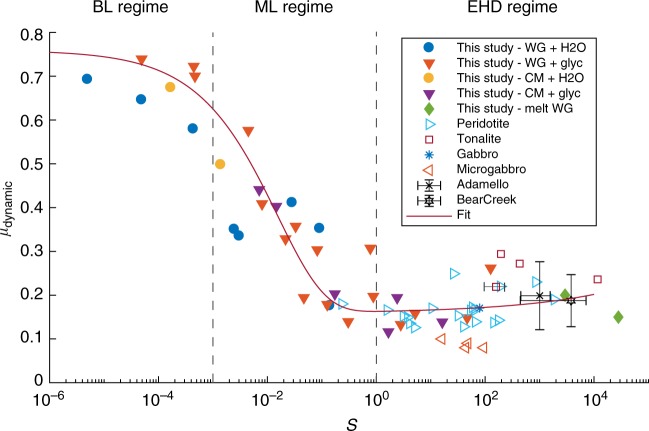
Dynamic friction coefficient vs. Sommerfeld number. Experiments performed in water and glycerol presented in this study are compared with previous experiments with evidence of frictional melting performed on peridotite^31^, tonalite^32^, gabbro^33^, and microgabbro^10^ (see symbols in the figure with WG for Westerly Granite and CM for Carrara Marble, see Supplementary Material Table 5). The viscosity of the melt for the room-humidity experiments were computed using the formulas proposed by Giordano et al.^50^. The *H*_0_ values were considered constant for the experiments performed in presence of melt and equal to 13 μm. The two black points with large error bars refer to field estimation of S from pseudotachylyte-bearing faults (Gole Larghe fault zone in Adamello (Italy) and Bear Creek fault zone in Mount Abbot Quadrangle (USA)), see Supplementary Note 1. For *S* < 10^−3^, boundary lubrication regime, the dynamic friction coefficient is almost independent of *S*. For 10^−3^ < *S* < 1, mixed lubrication regime, the dynamic friction coefficient decreases drastically with increasing *S*. For *S* > 1, elastohydrodynamic lubrication regime, the dynamic friction increases slightly with *S*. The best-fit curves are obtained using Eq. (1) (main text) with coefficients of determination of *R*^2^ = 0.90. The scattering in *µ*_dyn_ for *S* ≥ 1 is attributable to the viscosity of the fluid given that *τ* ∝ *ηV*

The *μ*_dyn_ varied with *S* (Fig. 2): for *S* < 10^−3^, *μ*_dyn_ was ~0.70 ± 0.05 and nearly independent of *S* (velocity neutral, or BL regime); at 10^−3^ < *S* < 1, *μ*_dyn_ decayed with *S* from ~0.7(*S* = 10^−3^) to ~0.2 (*S* = 1) (ML regime); at *S* > 1, *μ*_dyn_ slightly increased with increasing slip-rate (EHD regime). This Stribeck-type behaviour was similar for both water and water/glycerol mixtures but shifted to greater *S* values for the latter (Supplementary Figure 6). Importantly, the behaviour is similar for the two rock types suggesting that the obtained Stribeck curve relates to the rheology of the fluid mixtures rather than to the peculiar dynamic weakening mechanism of granite (flash and bulk melting^2^) or Carrara Marble (temperature and grain-size dependent^29^). Note that *μ*_dyn_ slightly decreased with increasing *V* as proposed by Di Toro et al.^2^, but was high scattered (Supplementary Figure 5).

Following the Stribeck model proposed by Canudas de Wit et al.^30^ and based on the solution on motion at so-called steady state conditions for metallic frictional interfaces, our friction data can be described by:

1 $$\mu _{{\mathrm{dyn}}} = \mu _{\mathrm{c}} + \left( {\mu _{{\mathrm{static}}} - \mu _{\mathrm{c}}} \right) \cdot e^{ - \beta \sqrt S } + \gamma \cdot S{,}$$

where *μ*_static_ (=0.7) is the Coulomb friction coefficient corresponding to *μ*_static_ under dry conditions (i.e., without fluid lubrication), *μ*_c_ (=0.1) is the friction coefficient at the full film lubrication condition (~*μ*_dyn_ during EHD), and *β* and *γ* are empirically determined linear coefficients. The second and the third right-hand terms of Eq. (1) depict, respectively, the Stribeck effect (the decrease in the friction coefficient due to an increase in *V*) and the viscous friction (the increase in the friction coefficient due to the presence of viscous fluids). We used a linear regression procedure to estimate the coefficients *β* and *γ* for the for the original experimental dataset presented here (Supplementary Table 2), plus published experimental and field data (from Eq. (1), coefficient of determination *R*^2^ = 0.90, *β* = 8.03, and *γ* = 0.004) (Fig. 2). In fact, this relation holds for other silicate-bearing rocks (gabbro, peridotite, etc.^10,31–33^) tested at seismic slip conditions, and where frictional melting occurred, highlighting the universality of EHD mechanism when fluids are present in the fault slipping zone (Fig. 2).

Microstructural investigation of the slipping zones (Supplementary Figure 2) recovered after the experiments performed on Westerly Granite at *V* = 1 m/s under room-humidity and water conditions (corresponding to *S* < 1), showed evidence of frictional melting^34^. Instead, there was no evidence of frictional melting in the slipping zones recovered from the experiments conducted on 85 and 99% of glycerol where the fully lubricated regime (*S* > 1) was achieved (Supplementary Figure 2). Moreover, for *S* > 1 the roughness of the slip surfaces at the end of the experiments was comparable to the initial roughness (Supplementary Figure 3). In the case of Carrara marble, the slipping zone after the experiments conducted at *V* > 0.1 m/s under either room humidity^9^ or water-flooded conditions when *S* < 1, was made of submicrometre in size (recrystallised) grains. Instead, there were no slipping zones made of submicrometre in size grains after the experiments conducted with 99% glycerol and at high slip-rate where the fully lubricated regime (*S* > 1) was achieved (Supplementary Figure 4). As was the case for the experiments performed on Westerly Granite, the sheared samples had a surface roughness very similar to the initial nonsheared one. In conclusion, microstructural observation and surface roughness measurements suggest that for *S* > 1 most of the shearing was accomodated within the glycerine-rich fluid, confirming the activation and effectiveness of EHD lubrication.

### Rupture propagation criteria and extrapolation to natural case

**Fig. 3:**
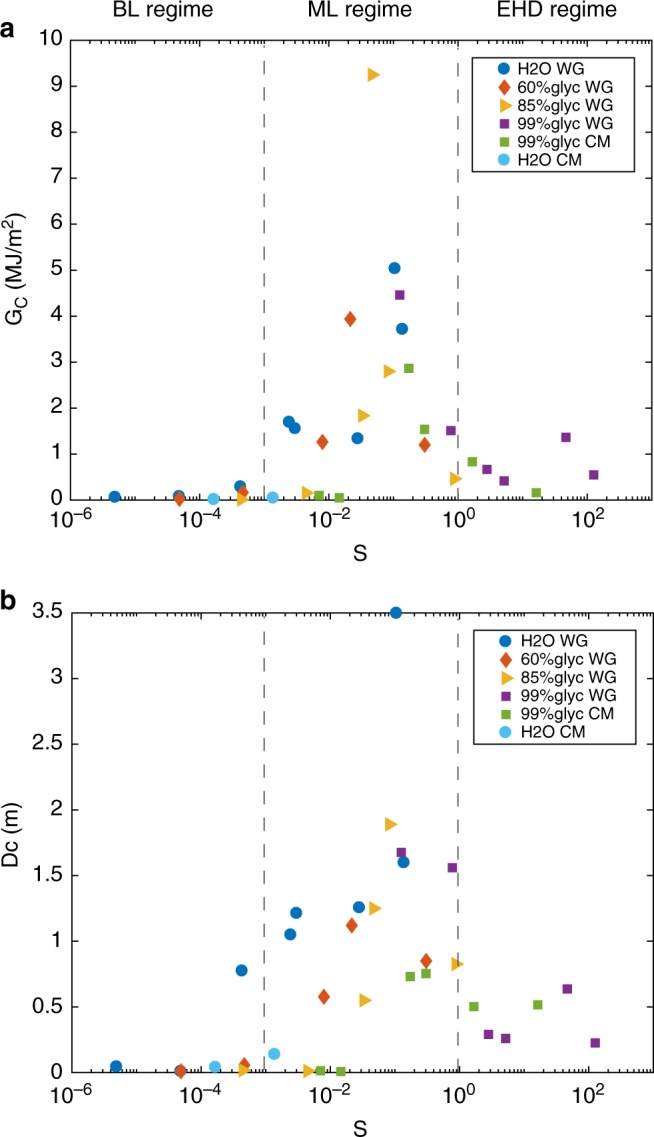
Fracture energy and *D*_c_ vs. Sommerfeld number. The three lubrication regimes (BL, ML, and EHD) are delimited by vertical black dashed lines. Experiments were performed at acceleration of 6.5 m s^−2^, effective normal stress *σ*_eff_ up to 20 MPa in the presence of water, mixtures of glycerol, and water and pure glycerol (see symbols in the figure where WG is Westerly Granite and CM is Carrara Marble. **a** Fracture energy vs. Sommerfeld number. **b** Slip weakening distance *D*_c_ vs. Sommerfeld number

We now briefly discuss to what degree the lubrication processes measured here may promote rupture propagation and earthquake slip in a faulting scenario. Increasing *S* reduced the dynamic friction coefficient (*μ*_dyn_) (Fig. 2); however, this will come at the cost of increasing the fracture energy (*G*_c_), at least in the BL and ML regimes (*S* > 1) (Fig. 3). Indeed, *G*_c_ increased exponentially with *S* in BL and ML regime, i.e., *S* < 1 up to 9 MJ m^−2^. Instead, at the boundary between the ML and the EHD regimes, *G*_c_ dropped sharply back to values of ~1 MJ m^−2^, evidencing the transition between ML and EHD regimes. Therefore, lubrication affects two competing mechanisms and does not necessarily promote dynamic earthquake rupture propagation.

To illustrate this, we may write a simplified rupture propagation criterion as

2 $$\frac{1}{2}\left( {\tau _0 - \tau _{{\mathrm{dyn}}}\left( S \right)} \right)\,U \ge G_{\mathrm{c}}\left( S \right){,}$$

where *τ*_0_ is the initial shear stress on the fault (whose upper bound is *τ*_static_ = *μ*_static_ *σ*_eff_), *τ*_dyn_ = *μ*_dyn_ *σ*_eff_), and where the *S* dependence of *τ*_dyn_(*S*) and *G*_*c*_(*S*) is made explicit. As seen in the left-hand term, the effectiveness of lubrication increases with the amount of fault slip *U*. A large value of *G*_c_ may prevent the dynamic propagation of smaller earthquakes, but as *U* increases the lubrication effect will become dominant. Because fracture energy will remain constant after the critical slip distance *U* = *D*_c_ (Fig. 1a), but the left-hand side of Eq. (2) will continue to increase with slip, the indicative value *U* ≈ *D*_c_ will mark the watershed between rupture-hindering and rupture-promotion by lubrication under intermediate *S* values. Note that when *S* > 1, both *G*_c_ and *μ*_dyn_ are small, favouring earthquake propagation.

While our experimental protocol allows us to impose and estimate the parameters (*η*, *V*, *σ*_eff_, *L*/*H*_0_) which control *S*, the same parameters are poorly known in nature. Here, below we attempt an estimate of the possible ranges of (*η*, *V*, *σ*_eff_, *L*/*H*_0_) for natural faults. Water is pervasive in the Earth's crust^8,35–37^: the viscosity of water ranges from ~1 mPa s at subsurface conditions (~1 km depth) to ~0.1 mPa s at a depth of 10 km (considering a thermal gradient of 30 °C/km and a linear increase in *σ*_eff_ with depth; Supplementary Figure 9). Alternatively, viscous fluids along faults may be produced by frictional melting during seismic slip^2^; a minimum value of 10 Pa s has been estimated^33^ for frictional melt viscosity. Moreover, especially in the shallower section (<5 km) of the seismogenic continental faults, fine grain gouge material mixed with water may also act as lubricant whose viscosity depends on the gouge grain size and the solid volume fraction. Viscosities of ~10 Pa s have been estimated for such fluid-saturated gouges^38–40^. Moreover, fluids commonly used in hydraulic fracturing operations in engineering reservoirs have viscosities ranging between 1 mPa s and 1 Pa s. As a result, the viscosity of fluids in the upper crust is estimated to range from 0.1 mPa s to more than 10 Pa s.

A value of 1 m/s is widely accepted^20^ as indicative of fault seismic slip-rate. The *L* parameter in *S* under EHD conditions corresponds to the longest (and dominant) wavelength in the mismatch between two rough sliding surfaces. In natural faults, *L* corresponds to the asperity wavelength, the longest of which is proportional to slip^4^ (and the magnitude) during a given earthquake. For earthquakes of magnitude ranging between 1 and 8, the average seismic slip and thus *L* = 1 mm–4 m^41^. Natural faults' surface roughness is almost self-similar^42^ with a ratio $\frac{L}{{H_0}}\sim 1000$: *H*_0_ is ranging between 0.001 to 4 mm for a M1 earthquake and M8 earthquake respectively. The lithostatic stress *σ*_1_ = *ρ*_*r*_*gz* (with *ρ*_*r*_ = 2700 kg/m^3^ rock density, *z* depth and *g* gravity) minus the hydrostatic water pressure (with *ρ*_*w*_ = 1000 kg/m^3^) at the earthquake hypocentral depth yields as an indicative value of *σ*_eff_ = (*ρ*_*r*_ − *ρ*_*w*_)*g z/K*, with $K = \frac{{1 \,+ \,{\mathrm{sin}}\left( \phi \right)}}{{1 \,- \,{\mathrm{sin}}\left( \phi \right)}}$, friction angle *ϕ* = arctan(*μ*), *μ* = 0.75, acting on the fault; this results in *σ*_eff_ ranging from ~50 MPa (at *z* = 3 km) to ~170 MPa (at *z* = 10 km).

The above hypothesis for the range of parameters (*V* = 4 m/s, *L* = 1 mm–4 m, *H*_0_~0.001–4 mm, *σ*_eff_ ≤ 170 Mpa, *η*(*P*, *T*) ofwater) would results in *S* values ranging from 5.0 × 10^−3^ to 1.0 × 10^−6^ at *z* = 10 km for Mw = 1 to Mw = 8 and from 70 to 0.07 at *z* ~ 0 km for Mw = 1 to Mw = 8 (Supplementary Figure 9). These ranges for *S* are comparable to the *S* value obtained in our experiments, implying that BL, ML and EHD regimes can occur in the Earth's crust, depending on fluid viscosity, slip-rate and earthquake nucleation depth (Supplementary Figure 10), While the Somerfield number remains undetermined in most natural faults, few of well-exposed seismogenic fault zones (Gole Larghe and Bear Creek fault zones, Supplementary Note 1 and Supplementary Table 3 for details) where large scale roughness, nucleation depth and co-seismic velocity have been estimated and the occurrence of co-seismic fluids is attested by the presence of pseudotachylytes (solidified friction melts produced during seismic slip) support the trend of dynamic friction indicated by our laboratory tests (Fig. 2). This geological evidence support the hypothesis that EHD can operate in some natural earthquakes.

We have experimentally demonstrated that fluid pressure and *η* are critical parameters that control slip dynamics in experimental faults and we suggest that similar effects should be expected in the case of both man-induced and natural earthquakes. In particular, highly viscous fluids (1) slightly reduce the static friction coefficient fostering fault reactivation and (2) trigger EHD lubrication during seismic slip. However, in our experimental results, for a large range of possible viscosity values of fault-permeating fluids, typical seismic slip-rates would result in intermediate values of the Sommerfeld number (10^−3^ < *S* < 1) and relatively large *D*_c_ values. As a consequence the fracture energy during seismic propagation of small events (*U* < *D*_c_) is relatively large, inhibiting seismic rupture propagation in favour of slow strain energy relaxation by stable creep (*S* and *D*_c_ would both decrease at low slip-rate). In contrast, the presence of increasingly viscous fluids decreases the fracture energy dissipated for large events, making the fault weaker. Therefore, our results suggest that in the presence of highly viscous fluids, rupture is expected to grow quasi-statically on the fault until the slip of the order of tens of cm is attained (until the distance *D*_c_ is overcome). While the effect of fluid pressure in earthquake rupture has been previously explored mostly in terms of thermal pressurisation and effective stress, we argue that the role of fluid viscosity is also important in understanding the dynamics of a lubricated fault system, with implications for rupture energy budget and dynamic weakening of both natural and man-induced earthquakes.

## Methods

### Experimental procedure

The experiments were performed with SHIVA (slow to high velocity apparatus)^21^, a rotary shear machines installed in Rome, on full cylinder samples of Westerly granite and Carrara marble. Target slip-rates (*V*) ranged from 10 µm/s up to 3 m/s, acceleration and deceleration ramps were imposed to 6.5 m/s^2^ and normal stresses were up to *σ*_*n*_ = 22.6 MPa. Mechanical data (axial load, torque, axial displacement and angular rotation) were acquired at a frequency between 250 Hz and 25 kHz, depending on the target slip-rate. Slip, slip-rate and shear stress were determined using the method outlined in Niemeijer et al.^43^ and Tsutsumi and Shimamoto^44^. All the experiments (Supplementary Table 1) with fluids were performed under drained condition (i.e., pore pressure remained constant during the experiments), following the procedure described by Violay et al.^9^. The pressurising system consisted of a pore fluid vessel and a membrane pump with a 30 cm^3^ fluid capacity, a pressure multiplier which imposes up to 15 MPa of fluid pressure, a pressure regulator and valves and pipes.

In Supplementary Figure 1 we show the evolution of the apparent friction coefficient *μ* vs. displacement for four different experiments performed with SHIVA under 10 MPa of effective normal stress (*σ*_eff_), in presence of different mixtures of distilled water and glycerol in order to have an initial viscosity *η*_0_ increasing of a factor ca. 10, with a fluid pressure *P*_*f*_ = 2.7 MPa. In the figure, the apparent friction coefficient is fitted following the exponential decay function proposed by Mizoguchi et al.^26^ $\mu = \mu _{{\mathrm{dyn}}} + \left( {\mu _{{\mathrm{peak}}} - \mu _{{\mathrm{dyn}}}} \right)e^{\frac{{ln\left( {0.05} \right)U}}{{D_{\mathrm{c}}}}}$ and allow us to calculate the weakening distance *D*_c_.

### Surface roughness measurements

The 3D arithmetic surface roughness was determined on the sliding surface of 20 or 25 mm in diameter cores of the starting samples and post-mortem samples of Westerly granite and Carrara Marble using an optical profilometer ContourGT-I 3D Optical Microscope, Bruker Nano surfaces Division. The scan of the surfaces was performed imposing an overlap of 20% between two adjacent areas. RMS (root-mean-square) and the stitched images of the samples surface of Westerly Granite after the experiments performed under room humidity condition and in presence of the four different fluids at slip-rate of 1 m/s are shown in the Supplementary Figure 2. The RMS of the starting sample of Westerly Granite was around 13 µm. Measurement of roughness of Carrara Marble starting sample had an RMS = 7.05 µm (Supplementary Figure 4a).

The scan of postmortem surfaces for experiments performed in presence of the same lubricant (glycerol 99), but slided at different slip-rate, had roughness of the same order of magnitude when *S* ≥ 1 (Supplementary Figure 4c, Supplementary Figure 4d), and roughness of two order of magnitude higher for *S* < 1 (Supplementary Figure 4b).

When the Sommerfeld number *S* was higher than 1, the RMS of the samples at the end of the experiments was of the same order of magnitude of the initial RMS. In the experiments performed at high slip-rate the presence of high-viscous lubricants prevented the formation of melt and debris on the surfaces of Westerly Granite and Carrara Marble samples.

### LuGre dynamic friction model

The LuGre dynamic friction model was proposed by Canudas de Wit et al.^30^ to describe friction forces as a function of slip-rate in the three lubrication regimes (boundary, mixed and elasto-hydrodynamic lubrication) in presence of viscous fluids. In this model, the behaviour of the asperities and/or the fluid during shearing is assimilated to the behaviour of some bristles whose deflection can be described as

3 $$\frac{{dz}}{{dt}} = v - \frac{{\left| v \right|}}{{g\left( v \right)}}z{,}$$

where *z* is the deflection of the bristles, *v* is the slip rate and at steady state *z*_ss_ = *g*(*v*)*sgn*(*z*). The friction generated from the bending of the bristles is

4 $$F = \sigma _0z + \sigma _1\frac{{dz}}{{dt}} + \sigma _2v{,}$$

where *σ*_0_ is the stiffness, *σ*_1_ a damping coefficient and *σ*_2_ a viscous coefficient.

According to the model, in mixed and hydrodynamic regimes, the friction force for constant slip-rate at steady state is given by:

5 $$F_{{\mathrm{ss}}} = g\left( v \right)sgn\left( v \right) + f\left( v \right){,}$$

where *g*(*v*) seizes Coulomb friction and the Stribeck effect and it can be written as $g\left( v \right) = F_{\mathrm{c}} + \left( {F_{\mathrm{s}} - F_{\mathrm{c}}} \right)e^{ - \left| {\frac{v}{{v_{\mathrm{s}}}}} \right|^\alpha }$, where *F*_s_ corresponds to the stiction force (i.e., the force necessary to start the motion), and *F*_c_ is the Coulomb friction force, i.e., the force necessary to continue sliding in absence of lubricants, *v* is the relative slip-rate between the moving solid bodies. The characteristic slip-rate of the Stribeck function *v*_*s*_ determines how quickly *g*(*v*) approaches *F*_c_ and depends on fluid viscosity and loading conditions. As suggested by Bo and Pavalescu^45^, the *α* parameter ranges between 0.5 and 1. Instead, *f*(*v*) is the viscous friction^46^ and typically is given in the form *f*(*v*) = *σ*_2_ · *v*^30,47^. In this work, we found $v_{\mathrm{s}} \propto \left( {\sigma _{{\mathrm{eff}}} \cdot H_0^2} \right)/\eta \,L$ and $\sigma _2 \propto \eta \,L/\left( {\sigma _{{\mathrm{eff}}} \cdot H_0^2} \right)$.

We adopted the LuGre model to fit our experimental data of *μ*_dyn_, defined as the shear stress divided by the effective normal stress *σ*_eff_ (Supplementary Figure 6), following the Eq. (1) where the coefficients *β* and *γ* are a function of the interaction between the rock-solid material and the molecules of the viscous fluid^48^.

All the parameters of the model are reported in Supplementary Table 2 and result in the best fit curves of Supplementary Figure 6.

### Thermal model and viscosity correction with temperature

The increase of temperature in the slip zone and wall rocks of the sheared bulk samples during frictional sliding weas estimated using a 2D FEA time dependent model for heat diffusion.

The model reproduced the sizes of the experimental sample, (i.e., 50 × 55 mm or diameter vs. height of the sample) with 1464 triangular mesh elements. Two different materials were used to simulate the slip zone and the bulk material. The slip zone was represented as a 13 μm-thick highly porous media. In agreement with the roughness measurements reported in Supplementary Figure 2 and Supplementary Figure 4, we considered *ϕ* = 1 − *A*_r_/*A* = 0.95 where *A*_r_ is the real contact area and *A* is the nominal area. The bulk material was regarded as a very low porous media (3% porosity, measured with the helium pycnometer). We assumed that all the mechanical energy is converted into heat and no heat is lost by radiation, so the heat flux *Q*(*r*, *t*) = 0.5 · *τ*(*t*)·*V*(*r*, *t*) is function of time *t* and the the radial distance *r* from the centre of the sample. A Neumann boundary condition was applied to the bottom external edge of the model (i.e., slip zone Supplementary Figure 5a). On the other three external boundaries, a constant temperature *T* = 293.15 K was imposed as the initial temperature of the two materials (energy dissipated by the steal of the pressure vessel). At the inner boundary between the slip zone and the wall-rock, the continuity of the solution was granted. The thermal properties of the slip zone were defined as a linear combination of the thermal properties of the fluid and of the rock which are reported in Supplementary Table 4. The thermal diffusivity of the slip zone:

6 $$\alpha _{{\mathrm{eff}}} = K_{{\mathrm{eff}}}/\left( {\rho \cdot C} \right)_{{\mathrm{eff}}}{.}$$

With *K*_eff_ = (1 − *ϕ*)·*K*_*r*_ + ϕ · *K*_*f*_ and (*ρ*·*C*)_eff_ = ((1 − *ϕ*)·*ρ*_*r*_·*C*_*r*_) + (*ϕ*·*ρ*_*f*_·*C*_*f*_) where *r* and *f* are related to the rock and fluid properties, respectively.

The diffusion of the heat is:

7 $$\left( {\rho \,C} \right)_{{\mathrm{eff}},{i}} \,\cdot \,\partial \frac{T}{\partial }t = \nabla \cdot \left( {\kappa _{{\mathrm{eff}},{i}}\,T} \right){,}$$

where *i* identified the two materials. The temperature in the slip zone was taken when the *μ*_dyn_ was reached (Supplementary Figure 7b).

Using the average estimated temperature in the slip zone at 2/3 *R* (*R* = 25 mm is the external radius of the sample, Supplementary Figure 7c), we corrected the initial viscosities (at 20 °C) of the fluids exploting the empirical law proposed by Cheng^49^ for water/glycerol mixtures.

The dynamic viscosity *η* of the mixture is:

8 $$\eta = \eta _{w}^\alpha \cdot \eta _{{\mathrm{glyc}}}^{1 - \alpha }{.}$$

Where *α* is weighting factor, function of the concentration of glycerol *C*_*m*_ and of two empirical factors *a* and *b* which are dependent of the temperature *T*

9 $$\alpha = 1 - C_{m} + \frac{{a\left( T \right) \cdot b\left( T \right) \cdot \left( {1 - C_{m}} \right)}}{{a\left( T \right) \cdot C_{m} + b\left( T \right) \cdot \left( {1 - C_{m}} \right)}}{.}$$

Supplementary Figure 8 shows the decrease of the initial fluids viscosities with increasing temperatures associated to frictional sliding.

### Rupture propagation criterion

The energy change *dW* [J/m] for an advancement *dL* of a crack of length *L* (if homogeneous conditions are assumed and radiated energy is neglected, which is a realistic assumption for the early phase of nucleation with slow rupture velocity) can be written as:

10 $$dW = - dL\,G_{\mathrm{c}} + \frac{1}{2}{\mathrm{\Delta }}\tau \,L\,dU{,}$$

where *dU* = *C*Δ*τ*/*μ*′*dL* is the slip increment, *μ*′ shear stiffness, Δ*τ* the stress drop and *G*_c_ the fracture energy.

Therefore we may write the energy delivery rate in a simplified form:

11 $$\frac{{dW}}{{dL}} = - G_{\mathrm{C}} + \frac{1}{2}\frac{{C{\mathrm{\Delta }}\tau ^2}}{{\mu \prime }}L{,}$$

with *C* a geometrical dimensionless constant.

For the crack to propagate the requirement is that $\frac{{dW}}{{dL}} \ge 0$, therefore

12 $$\frac{1}{2}C\frac{{\left( {{\mathrm{\Delta }}\tau } \right)^2}}{{\mu \prime }}L \ge G_{\mathrm{C}}{,}$$

substituting *L* with *U* by using *L* = *μ*′/(*C*Δ*τ*)*U* we obtain 1/2Δ*τ U* ≥ *C G*_c_ where the stress drop is Δ*τ* = *τ*_0_−*τ*_dyn_. Therefore we obtain:

13 $$\frac{1}{2}\left( {\tau _0 - \tau _{{\mathrm{dyn}}}} \right)U \ge G_{\mathrm{c}}{.}$$

## Supplementary information

Supplementary Information

Peer Review File

## Data Availability

Correspondence and request for additional material should be addressed to chiara.cornelio@epfl.ch. All the experimental raw data are available in Zenodo with the identifier “10.5281/zenodo.2557665”.
