# Supplementary material for: Mechanical behaviour of fluid-lubricated faults
Source: Nat Commun. 2019 Mar 20;10:1274. doi: 10.1038/s41467-019-09293-9 (PMC6426875; doi:10.1038/s41467-019-09293-9)
Supplement: Supplementary file 1 — Supplementary Information [file 41467_2019_9293_MOESM1_ESM.pdf]

Supplementary Information - Mechanical behavior of fluid lubricated faults.

Cornelio C. et al.

Supplementary Figures

Supplementary Figure 1

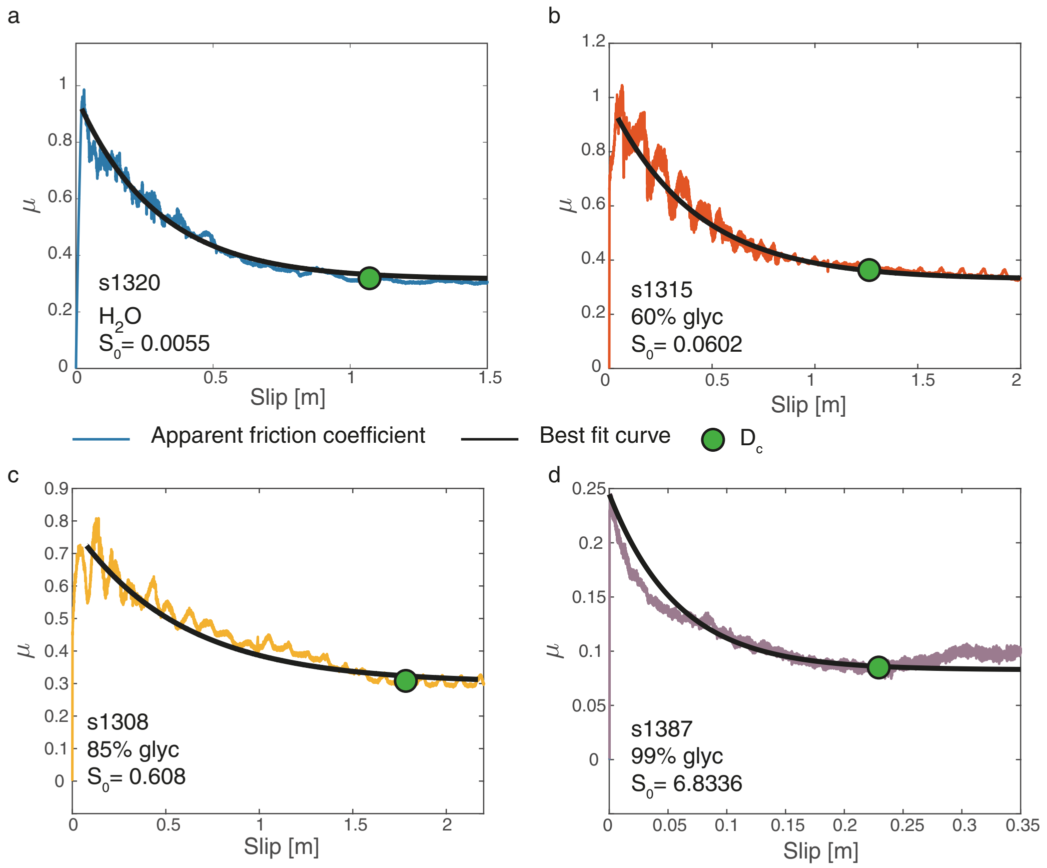

Supplementary Figure1: Apparent friction coefficient vs slip for experiments s1320, s1315, s1308, s1387 performed in presence of (a) water, (b) mixture 60%glycerol/40%water, (c) mixture 85%glycerol/15%water and d) pure glycerol in order to have a η_0 increasing of a factor $\sim$ 10 (i.e.Sommerfeld number S_0 at the target slip rate V increasing of a factor $\sim$ 10). The apparent friction coefficient is fitted by the exponential decay law proposed by Mizoguchi et al 2007[1], where D_c is the weakening distance. a) Test s1320 performed in presence of water ($\eta_0 = 1.002$ mPa s) at $P_f = 2.7$ MPa, $\sigma_{eff} = 10$ MPa, $V = 0.01$ m/s. b) Test s1315, performed in presence of mixture 60%glycerol/40%water ($\eta_0 = 10.8$ mPa s) at $P_f = 2.7$ MPa, $\sigma_{eff} = 10$ MPa, $V = 0.01$ m/s. (c) Test s1308, performed in presence of mixture 85%glycerol/15%water ($\eta_0 = 109$ mPa s) at $P_f = 2.7$ MPa, $\sigma_{eff} = 10$ MPa, $V = 0.01$ m/s. (d) Test s1387, performed in presence of pure glycerol ($\eta_0 = 1226$ mPa s) at $P_f = 2.7$ MPa, $\sigma_{eff} = 10$ MPa, $V = 0.01$ m/s.

Supplementary Figure 2

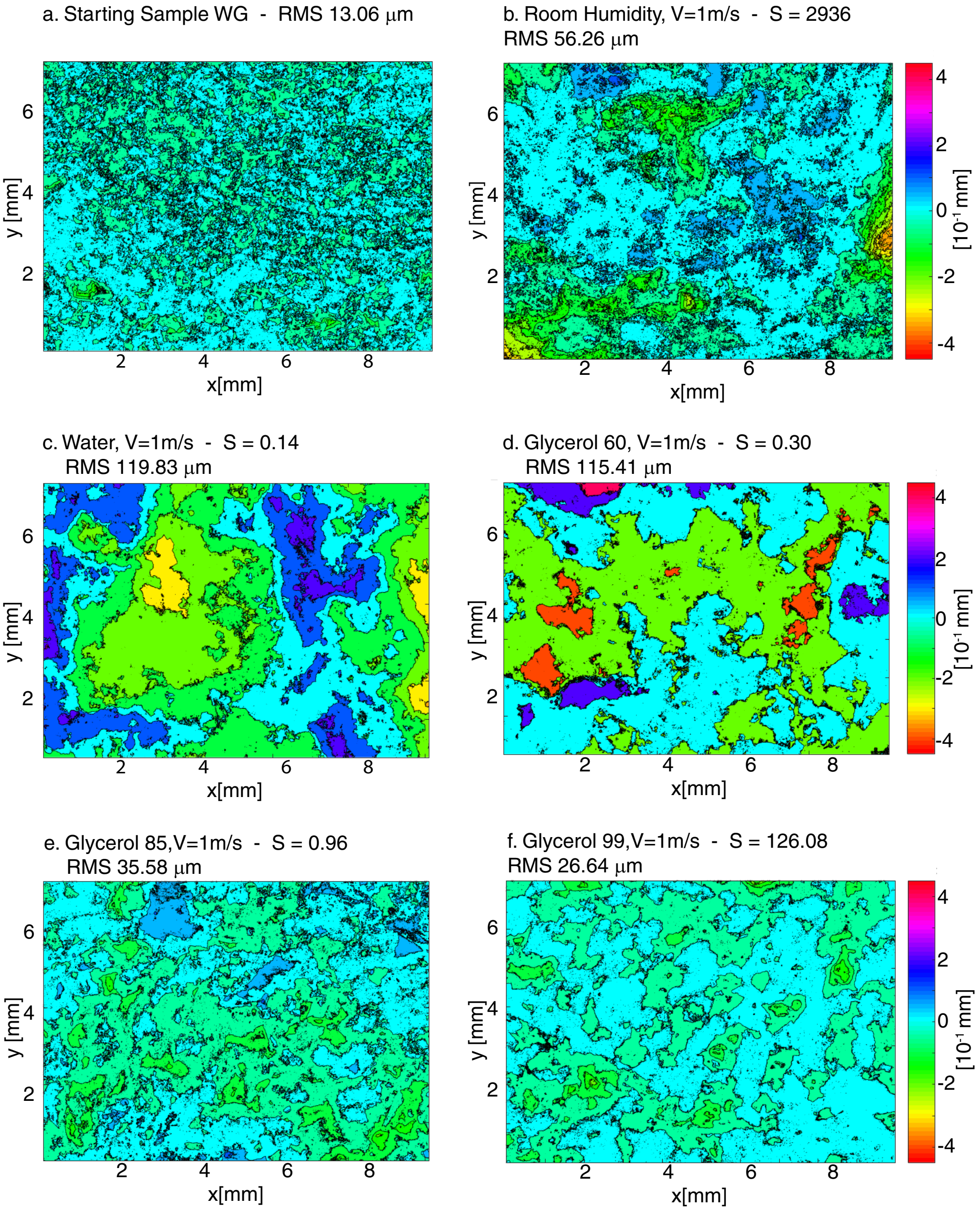

Supplementary Figure2: Roughness of the sliding surfaces and RMS values in Westerly Granite. (a) Starting sample and sheared samples under (b) room-humidity conditions (exp s1321), (c) water (exp s1303), (d) 60%glyc/40%water (exp s1316), (e) 85%glyc/15%water (exp s1305), and (f) 99%glyc (exp s1389). All the experiments were performed at $V = 1$m/s

Supplementary Figure 3

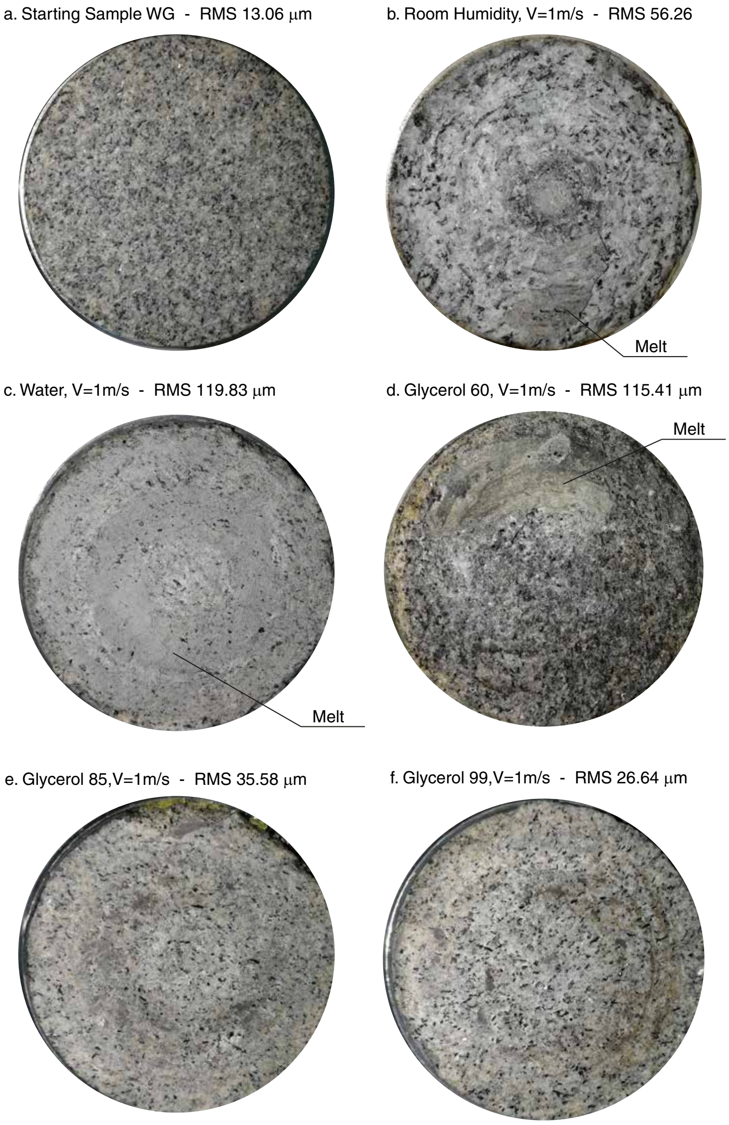

Supplementary Figure3: Microphotos of the sliding surfaces and RMS values in Westerly Granite. (a) Starting sample and sheared samples under (b) room-humidity conditions (exp s1321), (c) water (exp s1303), (d) 60%glyc/40%water (exp s1316), (e) 85%glyc/15%water (exp s1305), and (f) 99%glyc (exp s1389). All the experiments were performed at $V = 1\text{m/s}$.

Supplementary Figure 4

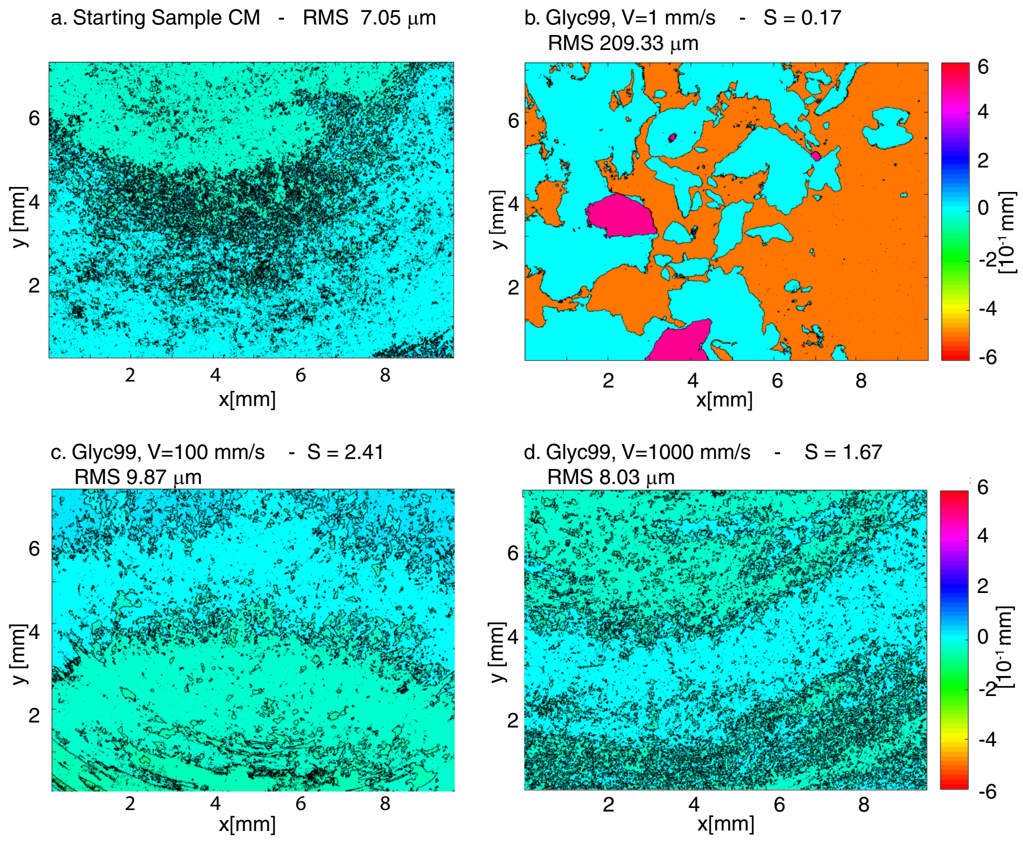

Supplementary Figure4: Roughness of the sliding surfaces and RMS values in Carrara Marble. (a) Starting sample, and samples immersed in 99%glyc sheared at (b) $V = 1$ mm/s (exp s1608), (c) $V = 100$ mm/s (exp s1607), (d) $V = 1000$ mm/s (exp s1606)

Supplementary Figure 5

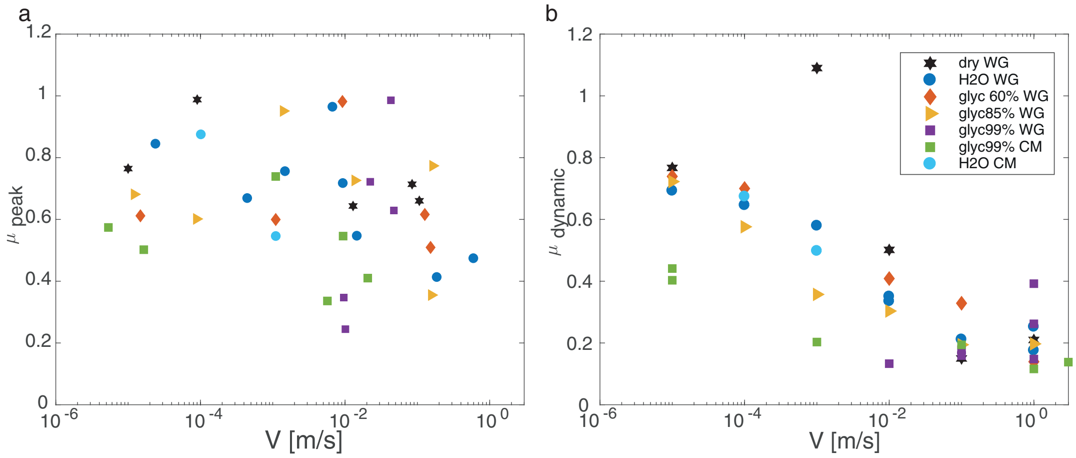

Supplementary Figure5: The (a)μ_{peak} and (b)μ_{dyn} vs slip-rate (V) for experiments performed in the presence of fluids and under room humidity conditions (dry, black stars). (a) Independently of the initial viscosity of the fluid μ_{peak} did not show a particular dependence with slip-rate with an average value of 0.75±0.14. (b) μ_{dyn} decreased with increasing V, but had a large scatter compared to its dependence with S (compare Supplementary Figure 5b with Supplementary Figure 6). At high V and only for experiments performed under room humidity conditions, we observed the formation of a viscous melt layer on the slip surfaces due to frictional heating. The similar trend for μ_{dyn} under room humidity and low viscosity pore fluid condition at high slip-rates is due to the formation of a continuous layer of melt.

7 # Supplementary Figure 6

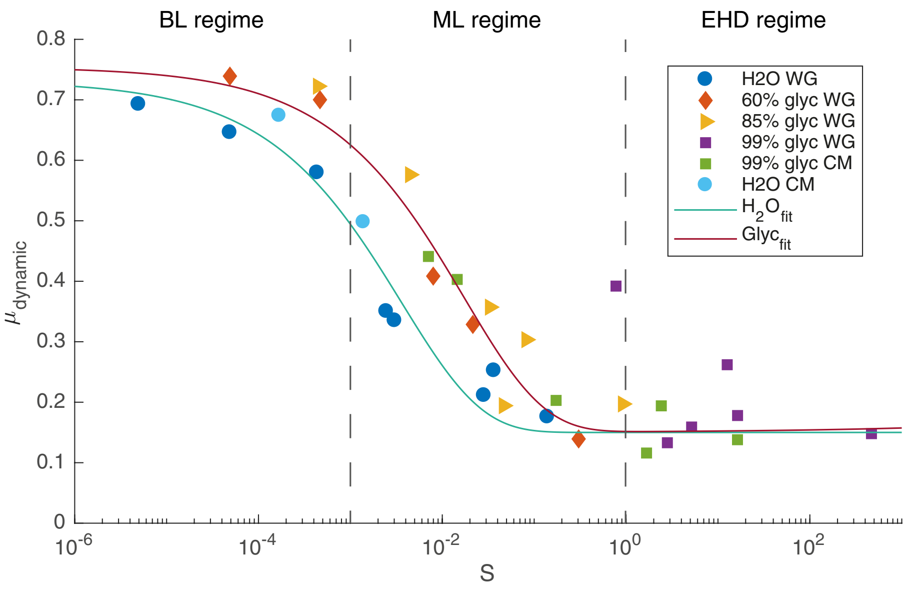

Supplementary Figure6: Dynamic friction coefficient versus Sommerfeld number S. Experiments were performed at acceleration of 6.5 m/s^2, effective normal stress σ_{eff} up to 20 MPa under the following environmental and hydraulic conditions: distilled water (H2O, blue dots for WG and cyan dots for CM) 60%glyc/40% water (orange diamonds), 85%glyc/15%water (yellow triangles) and pure glycerol (99%glyc, purple squares for WG and green squares for CM). For $S < 10^{-3}$, the dynamic friction coefficient decreases slightly with S. For $10^{-3} < S < 1$, the dynamic friction coefficient decreases dramatically with S. For $S > 1$, the dynamic friction increases slightly with S. The best fit curves were obtained using Eq.(2) and have coefficients of determination $R^2 = 0.95$ and $R^2 = 0.88$ for distilled water and glycerol mixtures, respectively.

Supplementary Figure 7

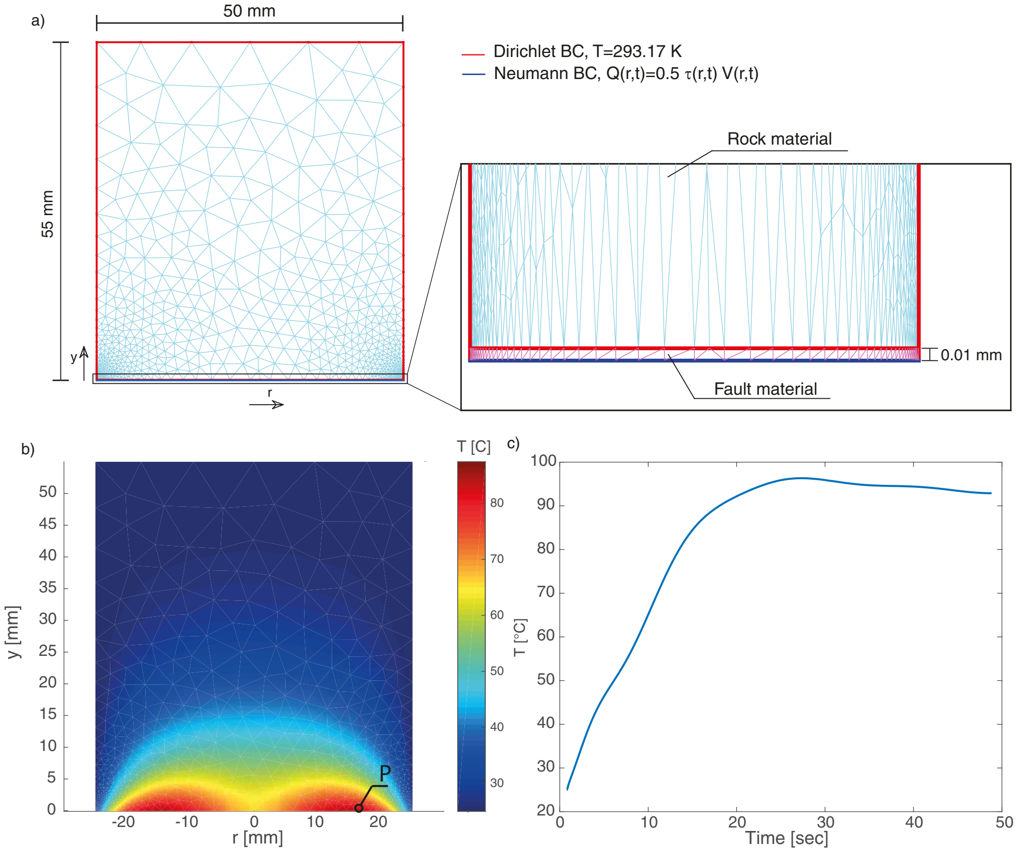

Supplementary Figure7: FEA 2D time dependent heat diffusion model. a) Mesh and Boundary Conditions of the model with zoom on the 0.01 mm-thick slip zone. b) Temperature distribution for the experiment s1315 at $t = 48$ s. c) Temperature evolution on the point P (x,y)=(0, 16.7 mm) for experiment s1315

9 # Supplementary Figure 8

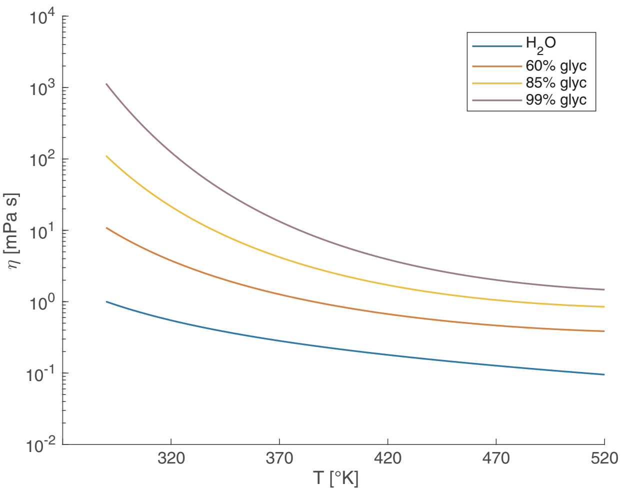

Supplementary Figure8: Evolution of fluid viscosities vs. temperature according to the empirical law of Cheng[2].

Supplementary Figure 9

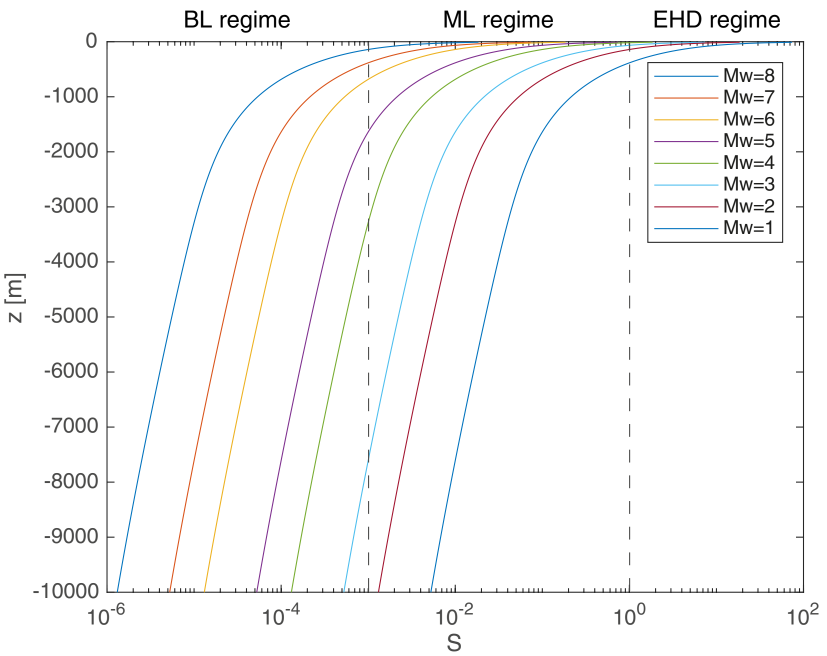

Supplementary Figure9: Sommerfeld number Vs depth for earthquakes magnitude ranging between 1 and 8.Considering the dependence of viscosity of water with temperature and pressure [3], we first estimated the decrease of viscosity from the surface to 10 km depth in the Earth's crust. We considered a surface temperature of 293.15 K, a temperature gradient of 30 K/km and a linear increase of pressure $\sigma_{eff} = ((\sigma_1 + \sigma_3))/2 + (\sigma_1 - \sigma_3))/2\cos(2\theta)$ with $\sigma_3' = \rho_r g z - \rho_f g z$, $\sigma_1' = K\sigma_3'$, $\theta = \pi/4 - \phi/2$, $\rho_r = 2700\ kg/m^2$ rock density, $\rho_f = 1/3\rho_r$, $g = 9.81\ m/s^2$, z depth in m, $K = (1 + \sin\phi)/(1 - \sin\phi)$, $\phi = \tan^{-1}\mu$, $\mu = 0.75$ friction coefficient. The estimated viscosity was used in $S = 6\eta V L/\sigma_{eff} H_0^2$ and we computed the decrease of S with depth for a range of earthquake magnitudes. In the computation of S, as a first approximation, we considered the relationship between earthquake magnitude and average slip on the fault (Table 2 of Sibson, 1989 [4]), a self-similar fault roughness (L/H 1000), and a constant slip-rate $V = 1$ m/s. According to the modeled dependence of S with depth, in the presence of water, EHD lubrication may occur for small in magnitude earthquakes and in shallow fault sections, consistently with the data reported in Fig. 2 of the main text. However, the presence of more viscous fluids (industrial fluids, friction melts) should extend the activation of EHD lubrication to deeper levels in the crust.

Supplementary Figure 10

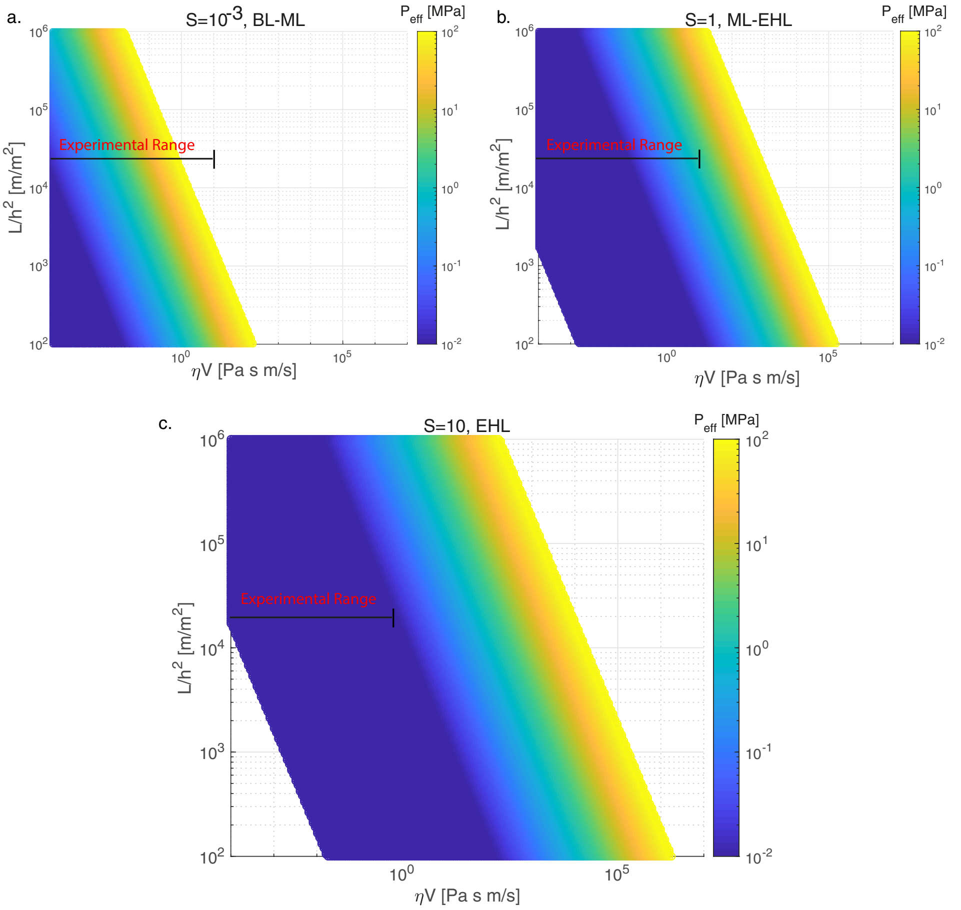

Supplementary Figure10: Range of values of η,V,L,H for which a.) S=0.001, limit between boundary lubrication regime and mixed lubricated regime; b.) S=1, limit between mixed lubrication regime and fully lubricated regime; c.) S=10, fully lubricated regime. The Sommerfield number is computed as $S = \dfrac{6\eta V L}{P_{eff} H^2}$ (see definition in the main text). The ratio h/L for natural fault ranges between 0.1% and 10% (see. Brodsky, 2016[5] and included references). Therefore the L/h^2 ranges between 100 mm^{-2} and 10^6 m m^{-2}. The fluid viscosity involved in natural earthquakes goes from η=1 mPa.s (for water) to η=10^3 Pa.s - 10^6 Pa.s (for melt), it depends of the temperature T of the slipping zone and the fluid composition. The slip-rate of V during earthquakes propagation ranges between 0.001 m/s and 10 m/s. Therefore the product $eta \cdot V$ ranges between 10^{-4} to 10^7 for natural cases. The effective pressure acting on a fault between 0 and 10 km depth ranges between 0 and 100 MPa (see Supplementary Figure S9). This figure clarifies that the range of possible values for the five parameters (L,h,V,η,P_{eff}) of the Sommerfield number in nature can create the conditions to have the three regimes also in natural fault zones. In particular, the EHL is likely to occur at surface for relatively low values of the ηV.

Supplementary Tables

Supplemtentary Table 1

Supplementary Table1: Summary of the experimental conditions and results. WG = Westerly Granite, CM = Carrara Marble, V=slip-rate, σ_{eff}=effective normal stress, η_0=initial kinematic viscosity of the fluid, μ_{static}=static friction coefficient, μ_{peak}= peak friction coefficient, μ_{dyn}= dynamic friction coefficient, G_c= fracture energy for m^2 fault, S= Sommerfeld number at μ_{dyn}

test	Conid.	V [mm/s]	σ_{eff} [MPa]	μ_{static} [-]	μ_{peak} [-]	μ_{dyn} [-]	D_c [mm]	G_C [MJ/m^2]	S
s1293	WG RH	0.01	10	0.661	0.765	0.767	20.00	0.039	-
s1399	WG RH	0.1	10	0.712	0.988	1.097	60.00	0.007	-
s1297	WG RH	10	10	0.530	0.644	0.501	901.06	0.609	-
s1301	WG RH	100	10	0.714	0.714	0.150	1801.90	6.293	2.80E4
s1321	WG RH	1000	10	0.660	0.660	0.210	1719.10	5.008	2.94E3
s1318	WG H2O	0.01	10	0.705	0.843	0.693	42.94	0.058	4.96E-6
s1319	WG H2O	0.1	10	0.636	0.667	0.646	7.80	7.366	4.88E-5
s1302	WG H2O	1	10	0.507	0.755	0.580	772.79	0.621	4.34E-4
s1320	WG H2O	10	10	0.438	0.963	0.350	1047.20	1.688	2.46E-3
s1394	WG H2O	10	10	0.418	0.716	0.335	1211.10	1.554	3.04E-3
s1386	WG H2O	100	10	0.390	0.546	0.211	1257.00	1.333	0.0284
s1303	WG H2O	1000	10	0.257	0.473	0.176	159.68	3.712	0.1393
s1393	WG H2O	1000	4	0.335	0.412	0.352	3461.00	5.026	0.0913
s1312	WG 60%gl	0.01	10	0.578	0.612	0.739	10.00	0.024	4.89E-5
s1313	WG 60%gl	0.1	10	0.507	0.600	0.700	57.00	0.163	4.67E-4
s1315	WG 60%gl	10	10	0.696	0.982	0.409	577.16	1.260	0.0080
s1317	WG 60%gl	100	10	0.588	0.616	0.329	1120.00	3.938	0.0215
s1316	WG 60%gl	1000	10	0.443	0.509	0.139	850.00	1.200	0.3048
s1306	WG 85%gl	0.01	10	0.488	0.681	0.723	18.00	0.011	0.0004
s1309	WG 85%gl	0.1	10	0.498	0.602	0.576	12.00	0.154	0.0045
s1304	WG 85%gl	1	10	0.686	0.951	0.357	550.00	1.836	0.0330
s1308	WG 85%gl	10	10	0.481	0.726	0.303	1890.00	2.801	0.0823
s1311	WG 85%gl	100	10	0.307	0.774	0.194	1250.00	9.247	0.0467
s1305	WG 85%gl	1000	10	0.315	0.355	0.197	826.00	0.462	0.9399
s1387	WG 99%gl	10	10	0.227	0.245	0.133	219.20	0.667	2.8149
s1388	WG 99%gl	100	10	0.346	1.240	0.159	261.10	0.417	5.1682
s1389	WG 99%gl	1000	10	0.248	0.722	0.392	1559.80	1.510	0.7770
s1390	WG 99%gl	1000	5	0.191	0.986	0.262	226.10	0.547	1.26E2
s1604	WG 99%gl	1000	5	0.293	0.347	0.148	637.25	1.363	46.7098
s1605	WG 99%gl	100	5	0.416	0.629	0.178	1676.12	4.467	0.1256
s1606	CM 99%gl	1000	10	0.353	0.546	0.116	502.50	3.346	1.6704
s1607	CM 99%gl	100	10	0.209	0.336	0.194	754.10	6.571	2.4175
s1608	CM 99%gl	1	10	0.425	0.739	0.203	731.10	8.870	0.1732

s1609	CM 99%gl	0.01	10	0.513	0.574	0.403	7.70	5.628	0.0145
s1610	CM 99%gl	0.01	20	0.371	0.502	0.441	13.93	0.184	0.0071
s1611	CM 99%gl	3000	5	0.331	0.410	0.138	516.63	0.159	16.3101
s1612	CM H2O	0.1	10	0.592	0.874	0.674	39.39	0.009	0.0002
s1613	CM H2O	1	10	0.429	0.545	0.498	137.19	0.039	0.0014

Supplementary Table 2

Supplementary Table2: Coefficients used in Supplementary Figure 6 for fit the data from experiments performed with water (H_2O fit) and glycerol mixtures (Glyc fit)

	μ_c	μ_s	β	γ	R^2
H2O fit	0.15	0.732	16.592	1.00E-05	0.95
Glyc fit	0.15	0.754	7.594	1.30E-03	0.88

Supplementary Table 3

Supplementary Table3: Parameters used for computing S and μ_{dyn} for natural exhumed faults with pseudotachylyte.

	σ_n [MPa]	τ_{ss} [MPa]	T [C]	η [Pa s]	L [m]	H [mm]	V [m/s]	S [$\cdot 10^3$]
Gole Larghe	112-182	14.9-48.1	1050-1450	4.79-389	0.1-3	34-168	1	1.01±0.55
Bear Creek	200-400	10.0-70.0	1000-1200	4.07-63.10	(0.01-1)E2	0.3-1	1	3.78±3.34

Supplementary Table 4

Supplementary Table4: Thermal properties of the fluid, Westerly granite and Carrara Marble. K=thermal conductivity, ρ= density, C= specific heat, WG=Westerly granite. CM=Carrara marble a) Thermal properties of water from Goranson, 1942[6], b) Thermal properties of water/glycerol mixtures from Bates, 1936[7] c) Thermal properties of Westerly granite from Eppelbaum et al 2014[8]

	H_2O^a	$60\%Glyc^b$	$85\%Glyc^b$	$99\%Glyc^b$	WG^c	CM^c
K [W m^{-1} K^{-1}]	0.6	0.3807	0.3096	0.2845	3.07	2.08
ρ [kg m^{-}3]	1000	1151	1219	1255.4	2650	2650
C [J kg^{-1} K^{-1}]	4180	3121.6	2678.1	2430	900	525

Supplementary Table 5

Supplementary Table5: Published experimental data reported in Fig.2 of the main text. σ_n=normal stress, V slip-rate, μ_{peak}= peak friction coefficient, μ_{dyn}= dynamic friction coefficient, T= estimated temperature at μ_{dyn}, η= melt estimated viscosity, S= Sommerfeld number, Ref.= paper references.

Rock	Run	σ_n [MPa]	V [m s^{-1}]	μ_{peak}	μ_{dyn}	T [°C]	η [Pa s]	S	Ref.
Peridodite	HVR616	15.00	1.14	0.67	0.17	1443	1.82	10.50	[9]
Peridodite	HVR617	20.00	1.14	0.73	0.18	2031	0.05	0.24	[9]
Peridodite	HVR615	10.00	1.14	0.62	0.22	1214	21.88	190.63	[9]
Peridodite	HVR618	5.00	1.14	0.8	0.23	1156	48.98	853.53	[9]
Peridodite	HVR620	13.00	1.14	0.50	0.13	1321	6.02	40.39	[10]
Peridodite	HVR623	15.59	1.14	0.40	0.15	1326	5.75	32.16	[10]
Peridodite	HVR633	16.13	1.14	0.60	0.17	1276	10.00	54.02	[10]
Peridodite	HVR634	7.78	1.14	0.63	0.17	1334	5.25	58.78	[10]
Peridodite	HVR635	5.37	1.14	0.52	0.19	1103	114.82	1863.00	[10]
Peridodite	HVR640	10.42	1.14	0.573	0.14	1295	7.94	66.42	[10]
Peridodite	HVR676	12.99	1.14	0.45	0.14	1200	26.30	176.43	[10]
Peridodite	HVR677	13.00	0.76	0.43	0.14	1186	32.36	144.60	[10]
Peridodite	HVR621	10.40	1.14	0.51	0.17	1299	7.76	65.04	[10]
Peridodite	HVR641	13.00	1.14	0.69	0.15	1594	0.56	3.77	[10]
Peridodite	HVR643	13.01	0.23	0.68	0.25	1222	19.50	26.35	[10]
Peridodite	HVR644	12.98	0.92	0.69	0.14	1553	0.75	4.11	[10]
Peridodite	HVR645	13.01	0.76	0.65	0.17	1660	0.35	1.58	[10]
Peridodite	HVR651	13.00	1.14	0.69	0.15	1621	0.47	3.14	[10]
Peridodite	HVR652*	13.02	1.14	0.61	0.13	1551	0.76	5.08	[10]
Tonalite	HVR373	15.00	1.14	0.65	0.27	1377	74.13	430.62	[9]
Tonalite	HVR375	10.00	1.14	0.80	0.29	1499	22.39	195.07	[9]
Tonalite	HVR377	20.00	1.14	0.50	0.22	1447	36.31	158.18	[9]
Tonalite	HVR379	15.00	1.20	0.40	0.24	1126	1905.46	11651.22	[9]
Microgabbro	s555	20.00	3.00	0.68	0.09	1249	3.38	47.88	[11]
Microgabbro	s563	20.00	1.00	0.59	0.1	1250	3.39	15.96	[11]
Microgabbro	s567	25.00	3.00	0.55	0.08	1149	6.61	93.36	[11]
Microgabbro	s585	20.00	3.00	0.57	0.08	1221	3.09	43.67	[11]
Gabbro	HVR687	15.50	1.14	0.65	0.17	1338	14.13	79.22	[12]

Supplementary Note 1

Pseudotachylyte is the result of solidification of friction-induced melts produced during seismic slip. Here we consider two well exposed pseudotachylyte-bearing faults, estimate the coseismic S and compare these natural cases with the experimental results.

Case A: Gole Larghe fault zone (Adamello, Southern Italian Alps)

The normal stress acting on the sub-vertical faults at 10 km depth was estimated range between 112 and 182 MPa [13, 9] and the dynamic shear stress between 14.9 and 48.1 MPa [14]. These values yielded a dynamic friction coefficient between 0.4 and 0.08, well below the typical coefficient for tonalites (0.7). The microstructures found in the pseudotachylyte were consistent with temperature during seismic sliding ranging from 1050 to 1450 °C. The chemical composition of the pesudotachylyte matrix is reported in [15]. Using the viscosity model for magmatic liquid proposed by Giordano et al. [16], we estimated the pseudotachylyte viscosity during sliding ranging between 4.79 and 389 Pa s. Griffith et al. [17] measured the 2D roughness of the pseudotachylyte wall rock boundary in two fault profiles. They identified $L = 0.1 - 3$ m, a Hurst exponent h ranging between 0.4 and 0.47 and a roughness amplitude factor $\beta \approx 0.1$. Based on Griffith et al. measurements, we computed $H = \beta L^h = 0.034 - 0.168$m. The above data yielded a value of $S = (1.01 \pm 0.55)$E3 (Supplementary Table 3)

Case B: Bear Creek fault (Mount Abbot Quadrangle, central Sierra Nevada)

Ague and Brimhall [18] estimated the initial normal stress acting on the fault ranging between 200 and 400 MPa. Considering a stress drop on the fault between $\Delta\sigma = 90 - 250$ MPa [19] and a initial shear stress of $0.65 - 0.8\,\sigma_n$, we can estimate a $\tau_{dyn} = (0.6 - 0.85)\,\sigma_n - \Delta\sigma$. The microstructures found in the pseudotachylyte matrix were consistent temperatures during seismic slip ranging from 1000 to 1200 °C. The chemical composition of the pseudotachylyte matrix is reported in [20]. Using the viscosity model for magmatic liquid proposed by Giordano et al. [16], we estimated the pseudotachylytes viscosity during sliding between 4.07 and 63.1 Pa s. From the study of Griffith et al. [17] the wavelength of the pseudotachylyte-wall rock boundary ranged between $L = 0.0001 - 0.01$ m, the Hurst exponent $h = 0.4 - 1$, so the asperity size is ranging between 0.0003 and 0.001 m. The above data yielded a value of $S = (3.78 \pm 3.34)$E3 (Supplementary Table 3).

Supplementary References

[1] Mizoguchi, K., Hirose, T., Shimamoto, T. & Fukuyama, E. Reconstruction of seismic faulting by high-velocity friction experiments : An example of the 1995 Kobe earthquake. *Geophys.*

Res. Lett. **34** (2007).

[2] Cheng, N.-s. *Industrial and. Engineering Chemistry Research* .

[3] IAPWS. Revised Release on the IAPWS Formulation 2008 for the Viscosity of Ordinary Water Substance. Tech. Rep., Berlin, Germany (2008).

[4] Sibson, R. H. Earthquake faulting as a structural process. *J. Struct. Geol.* **11**, 1–14 (1989).

[5] Brodsky, E. E., Kirkpatrick, J. D. & Candela, T. Constraints from fault roughness on the scale-dependent strength of rocks. *Geology* **44**, 19–22 (2016).

[6] Goranson, R. W. Heat Capacity; Heat of Fusion. In Chairman, F. B., Schairer, J. F. & Spicer, H. C. (eds.) *Handb. Phys. Constants* (Geological Society of America, 1942). URL `http://dx.doi.org/10.1130/SPE36-p223`.

[7] Bates, O. K. Thermal Conductivity of Liquids: Binary Mixtures of Water and Glycerol. *Ind. Eng. Chem.* **28**, 494–498 (1936).

[8] Eppelbaum, L., Kutasov, I. & Pilchin, A. *Thermal properties of Rocks and Density of Fluids* (2014). URL `http://link.springer.com/10.1007/978-3-642-34023-9`.

[9] Di Toro, G., Hirose, T., Nielsen, S. & Shimamoto, T. Relating high-velocity rock-friction experiments to coseismic slip in the presence of melts. *Geophys. Monogr. Ser.* **170**, 121–134 (2006).

[10] Del Gaudio, P. *et al.* Frictional melting of peridotite and seismic slip. *J. Geophys. Res.* **114** (2009).

[11] Violay, M. *et al.* Effect of water on the frictional behavior of cohesive rocks during earthquakes. *Geology* **42**, 27–30 (2014).

[12] Nielsen, S., Di Toro, G., Hirose, T. & Shimamoto, T. Frictional melt and seismic slip. *J. Geophys. Res.* **113**, 1–20 (2008).

[13] Di Toro, G. & Pennacchioni, G. Fault plane processes and mesoscopic structure of a strong-type seismogenic fault in tonalites (Adamello batholith, Southern Alps). *Tectonophysics* **402**, 55–80 (2005).

[14] Di Toro, G., Pennacchoni, G. & Nielsen, S. *Fault zone properties and earthquake rupture dynamics*, 87–133 (2009).

[15] Di Toro, G. & Pennacchioni, G. Superheated friction-induced melts in zoned pseudotachylytes within the Adamello tonalites (Italian Southern Alps). *J. Struct. Geol.* **26**, 1783–1801 (2004).

[16] Giordano, D., Russell, J. K. & Dingwell, D. B. Viscosity of magmatic liquids: A model. *Earth Planet. Sci. Lett.* **271**, 123–134 (2008).

[17] Griffith, W. A., Nielsen, S., Di Toro, G. & Smith, S. A. Rough faults, distributed weakening, and off-fault deformation. *J. Geophys. Res. Solid Earth* **115**, 1–22 (2010).

[18] Ague, J. J. & Brimhall, G. H. Magmatic arc asymmetry and distribution of anomalous plutonic belts in the batholiths of California: Effects of assimilation, crustal thickness, and depth of crystallization. *Bull. Geol. Soc. Am.* **100**, 912–927 (1988).

[19] Griffith, W. A., Toro, G. D., Pennacchioni, G., Pollard, D. D. & Nielsen, S. Static stress drop associated with brittle slip events on exhumed faults. *J. Geophys. Res. Solid Earth* **114**, 1–30 (2009).

[20] Griffith, W. A., Di Toro, G., Pennacchioni, G. & Pollard, D. D. Thin pseudotachylytes in faults of the Mt. Abbot quadrangle, Sierra Nevada: Physical constraints for small seismic slip events. *J. Struct. Geol.* **30**, 1086–1094 (2008).
