# Supplementary material for: Mechanical behaviour of fluid-lubricated faults
Source: Nat Commun. 2019 Mar 20;10:1274. doi: 10.1038/s41467-019-09293-9 (PMC6426875; doi:10.1038/s41467-019-09293-9)
Supplement: Supplementary file 2 — Peer Review File [file 41467_2019_9293_MOESM2_ESM.pdf]

Editorial Note: This manuscript has been previously reviewed at another journal that is not operating a transparent peer review scheme. The manuscript was considered suitable for publication without further review at Nature Communications.
